# The Spiritual Well-Being Scale: Psychometric Evaluation of the Shortened Version in Czech Adolescents

**DOI:** 10.1007/s10943-016-0318-4

**Published:** 2016-10-27

**Authors:** Klara Malinakova, Jaroslava Kopcakova, Peter Kolarcik, Andrea Madarasova Geckova, Iva Polackova Solcova, Vit Husek, Lucie Kluzova Kracmarova, Eva Dubovska, Michal Kalman, Zuzana Puzova, Jitse P. van Dijk, Peter Tavel

**Affiliations:** 1 Olomouc University Social Health Institute, Palacky University in Olomouc, Univerzitni 244/22, 771 11 Olomouc, Czech Republic; 2 Graduate School Kosice Institute for Society and Health, Safarik University, Kosice, Slovak Republic; 3 Department of Health Psychology, Medical Faculty, Safarik University, Kosice, Slovak Republic; 4 Institute of Active Living, Faculty of Physical Culture, Palacky University in Olomouc, Olomouc, Czech Republic; 5 Institute of Psychology, The Czech Academy of Science, Prague, Czech Republic; 6 Department of Community and Occupational Medicine, University Medical Center Groningen, University of Groningen, Groningen, The Netherlands

**Keywords:** Shortened SWBS, Adolescents, Spirituality, Religiosity, Psychometric evaluation

## Abstract

The aim of this study was to psychometrically evaluate the shortened version of the Spiritual Well-Being Scale (SWBS) in Czech adolescents. A nationally representative sample of 4217 adolescents participated in the 2014 Health Behaviour in School-aged Children survey. The internal consistency of the SWBS was assessed using Cronbach’s alpha (*α*) and Mean Inter-Item Correlation (MIIC) values. The factor structure was evaluated using principal component analyses. After adjustment, our new seven-item version of the scale supports a two-factorial model of the SWBS with satisfactory internal consistency (*α* = 0.814, MIIC = 0.379). This version of the SWBS is suitable for measuring spiritual well-being in a secularising environment.

## Introduction

Spirituality is a multidimensional construct (Hooker et al. 2014); therefore, its many definitions differ according to the dimension being emphasised by the authors. The concept of spirituality has been changing over time. Originally, it was connected with religiosity, but during the last decades its meaning has been further extended and has started to include additional concepts, such as purpose and meaning in life, connectedness with others, peacefulness, harmony and well-being (Koenig 2008).

There is a growing body of the literature that recognises the importance of both spirituality and religiosity and their possible role in physical and mental health (e.g. Aldwin et al. 2014; Hill and Pargament 2003; Weber and Pargament 2014). Therefore, the need for having effective ways of measuring spirituality is increasing. Meezenbroek et al. (2012) and Koenig (2008) both mentioned a high number of different spirituality questionnaires. One of the most extensively studied measures of subjective and spiritual well-being is the Spiritual Well-Being Scale (SWBS) (Ellison and Smith 1991; Koenig 2008; Paloutzian and Ellison 1982). The SWBS measures spiritual well-being, while distinguishing between its two interrelated yet distinct aspects: religious and existential well-being. The vertical dimension, Religious Well-Being (RWB), focuses on one’s relationship to God, while the horizontal dimension, Existential Well-Being (EWB), emphasises the sense of life purpose and life satisfaction (Ellison 1983).

Many studies have focused on spirituality among adults (e.g. Lawler-Row and Elliott 2009; Unterrainer et al. 2010), but fewer on adolescents. Consequently, also the number of suitable tools for measuring adolescent spirituality is more limited. One of the possible instruments could be the shortened version of the SWBS, as used by Cotton et al. (2005).

Spiritual development is a part of psychosocial and cognitive development (Sifers and Warren 2012). It is important to have a closer look at the possible protective role of spirituality regarding adolescent risk behaviour. Spirituality is of special interest in the conditions prevailing in the Czech Republic, as 76.4 % of the population is religiously unaffiliated (Pew Research Center 2014). Therefore, it is important to explore and clarify the problem of measuring adolescent spirituality under conditions of a highly secular society. The aim of this study is to psychometrically evaluate the shortened version of the SWBS in Czech adolescents.

## Methods

### Participants and Procedure

We obtained data on a nationally representative sample of Czech boys and girls from the 2014 Health Behaviour in School-aged Children study. Schools were selected randomly after stratification by region, school size and type of school (primary schools vs. secondary schools). Out of 243 contacted schools 242 schools agreed to participate (response rate 99.6 %). Then, classes from 5th, 7th and 9th grades, in general corresponding to age categories of 11-, 13-, and 15-year-olds, were selected at random, one from each grade per school. Data from 14,539 pupils were obtained (response rate 89.2 %). The majority of non-response was due to illness or other reasons, e.g. sports or academic competitions (10.6 %) and 30 children refused to participate in the survey (0.2 %).

Our study was restricted to half of the adolescents from the seventh and ninth grades who had the SWBS included in the questionnaire. This reduced the sample size to 4889. Because of incomplete information on age, gender or any of the responses on SWBS items, 672 questionnaires were excluded, leading to a final sample of 4217 respondents (mean age = 14.4, 48.8 % boys).

Data were collected between April and June 2014. The questionnaires were distributed by trained administrators while the teachers were not present in the classroom to reduce the response bias. Respondents had one school lesson (45 min) dedicated to completing the questionnaire. Participation in the survey was anonymous and voluntary. The study design was approved by the Ethics Committee of the Faculty of Physical Culture, Palacky University in Olomouc.

### Measures

The SWBS was translated from English by two independent Czech native speakers. Both versions were subsequently discussed in a working group of translators and researchers in order to create one tool. This was afterwards translated using the back-translation method by a professional native English translator, fluent in Czech, and compared with the original SWBS. After agreeing on the final version, the item clarity and understanding were tested on a focus group with satisfactory results.

The SWBS is composed of twenty items and measures two dimensions of spiritual well-being (Paloutzian and Ellison 1982). The Religious Well-Being (RWB) Subscale provides a self-assessment of one’s relationship with God, while the Existential Well-Being (EWB) Subscale gives a self-assessment measure of one’s sense of life purpose and life satisfaction. Each item is answered on a 6-point Likert scale ranging from “strongly agree” (1) to “strongly disagree” (6). Eight items are worded in a reverse direction and were reversely scored. The overall score from the SWBS is computed by summing the responses to all twenty items after reversing the negatively worded items. It ranges from 20 to 120, with a higher score representing greater spiritual well-being. For specific purposes, e.g. focusing only on one’s relationship with God or only on the existential well-being, the authors also admit the usage of only one subscale. For the purpose of this study the shortened version of the SWBS was used. According to Cotton et al. (2005), the number of items was reduced to 10, 5 of them belonging to the RWB and 5 to the EWB. In their study, the authors report a good internal consistency of the shortened scale with Cronbach’s *α* = 0.87. The total score of this shortened version ranges from 10 to 60. Of the total, 3 items are worded in a reverse direction.

Religiosity was measured by the frequency of attending church or religious sessions (religious attendance). The wording of the question was “How often do you go to church or to religious sessions?” with possible answers: several times a week/approximately once a week/approximately once a month/a few times a year/never. Those who reported attending religious sessions at least once a week were considered as attending.

### Statistical Analyses

Firstly, descriptive analyses for the study sample were performed. The Chi-square test and Mann–Whitney U test (2 groups) were used to test for statistical significance of gender differences in spiritual well-being (SBW, RWB and EWB) and church attendance. As a second step we calculated internal consistency indicators—Cronbach’s alpha (α) and Mean Inter-Item Correlation (MIIC)—for the whole SBWS as well as for the RWB and EWB subscales. As a third step we conducted an exploratory factor analyses (FA) with principal component analyses (PCA) and oblique rotation. Items with high shared loadings were deleted item by item, and in every step the internal consistency and factor structure were recalculated. The procedure was stopped when we reached satisfactory internal consistency and low shared loadings (lower than 0.15). Only the initial and final models are herein presented. All analyses were performed using the statistical software package IBM SPSS version 21.

## Results

As you might see in Table 1, boys showed significantly higher existential and overall spiritual well-being than girls, while there were no gender differences according to religious well-being and church attendance.

**Table 1:** Descriptive analyses of the shortened version of the SWBS of Czech adolescents for the whole sample and by gender

	Total (*n* = 4217)	Boys (*n* = 2056)	Girls (*n* = 2161)	*p* value
Church attendance: *n* (%)				.114^a^
Attending	302 (7.2)	134 (6.5)	168 (7.8)	
Not attending	3915 (92.8)	1922 (93.5)	1993 (92.2)	
SWB score: Mean (SD)	36.0 (7.73)	36.5 (7.63)	35.6 (7.80)	.000^b^
RWB score: Mean (SD)	13.2 (5.76)	13.3 (5.91)	13.1 (5.60)	.488^b^
EWB score: Mean (SD)	22.8 (4.93)	23.2 (4.85)	22.4 (4.97)	.000^b^

SD standard deviation; ^a ^Chi-square test; ^b ^Mann-Whitney U test

The initial visual inspection of data showed an unexpected abnormal shape, especially in the RWB histogram, with a solitary peak at the exact value of 10. This score was obtained by more than one-quarter of all respondents. A closer look showed a response pattern that pointed to a possible problem with the negatively worded item 5 (“I don’t get much personal strength and support from God”). Also a more detailed inspection of the two remaining negative EWB items (item 1 and 8) showed their major influence on the abnormal distribution of the EWB subscale.

The ten questions designed to assess the degree of overall spiritual well-being had relatively low internal consistency (*α* = 0.633, MIIC = 0.153). The RWB (*α* = 0.726, MIIC = 0.374) and EWB (*α* = 0.643, MIIC = 0.268) subscales showed slightly better internal consistency.

As a next step, an exploratory factor analysis with principal component analyses was employed. The scale’s developers used Varimax rotation (Ellison 1983), as well as some other researchers (Fernander et al. 2004; Miller et al. 1998). Other authors (Ledbetter et al. 1991), however, argue that because of the correlation between RWB and EWB subscales, an oblique rotation is more appropriate. For comparison purposes both Varimax and Oblimin rotations were performed and showed only negligible differences; therefore, only the results of the Oblimin rotation are presented. The initial solution yielded three potential factors with an eigenvalue higher than one, but the scree plot indicated only a two-factor solution. The test for legitimacy of the factor analysis resulted in the following coefficients: determinant of the correlation matrix = 0.012, Kaiser–Meyer–Olkin (KMO) measure of sampling adequacy = 0.825 and Bartlett’s test of sphericity (*p* < 0.001). The initial analysis with ten items yielded the three-factor solution shown in Table 2. The factor loadings revealed that items 2, 3, 6 and 9 constitute Factor 1, which corresponds with the RWB subscale, while items 4, 7 and 10 constitute Factor 2, which corresponds with the EWB subscale. The remaining three items (1, 5 and 8) created a third factor not proposed by the authors of the scale. A closer inspection of this factor showed that it was formed by the three negatively formulated statements. This three-factor solution explained 68.5 % of the overall variance.

**Table 2:** Factor structure of the shortened version of the Czech SWBS using Oblimin rotation

Items		Factor 1 (RWB)	Factor 2 (EWB)	Factor 3 (NFS)
6	I believe that God is concerned about my problems	**.920**	.197	−.249
3	I have a personally meaningful relationship with God	**.915**	.152	−.280
9	My relationship with God contributes to my sense of well-being	**.902**	.157	−.270
2	I believe that God loves me and cares about me	**.891**	.233	−.220
7	I feel good about my future	.136	**.845**	.039
4	I feel very fulfilled and satisfied with my life	.152	**.836**	.099
10	I believe there is some real purpose for my life	.222	**.760**	.054
8	Life doesn’t have much meaning	−.180	.235	**.769**
1	I don’t know who I am, where I came from, or where I’m going	−.188	.132	**.749**
5	I don’t get much personal strength and support from God	−.243	−.229	**.532**

*NFS* negatively formulated statements

The negatively formulated items were the same items that had high shared loading and were problematic regarding internal consistency, so we decided to delete them item by item. In every step, the internal consistency and the factor structure were recalculated. The procedure was stopped when we reached satisfactory internal consistency and low shared loadings (lower than 0.15). Finally, items 1 and 8 (belonging to EWB) and item 5 (belonging to RWB) were excluded. After excluding those items the internal consistency of the subscales increased remarkably for the RWB (*α* = 0.928; MIIC = 0.765) and slightly for the EWB (*α* = 0.760; MIIC = 0.516). The overall internal consistency for the new seven-item scale increased to *α* = 0.814 with MIIC = 0.379. After deleting all three items, we applied once more the test for legitimacy of the factor analyses, again with satisfactory results (determinant of the correlation matrix = 0.017, KMO = 0.822 and Bartlett´s Test of Sphericity *p* < 0.001). The recalculated factor analyses resulted in a two-factor solution (shown in Table 3) that explained 76.3 % of the overall variance.

**Table 3:** Factor structure of the shortened version of the Czech SWBS with the deleted items using Oblimin rotation

Items	Factor 1 (RWB)	Factor 2 (EWB)
3 I have a personally meaningful relationship with God	.920	
6 I believe that God is concerned about my problems	.916	
9 My relationship with God contributes to my sense of well-being	.903	
2 I believe that God loves me and cares about me	.890	
7 I feel good about my future		.852
4 I feel very fulfilled and satisfied with my life		.842
10 I believe there is some real purpose for my life		.774

Factor loadings smaller than 0.29 were suppressed

The original English language Spiritual Well-Being Scale (SWBS) is in the *Journal of Psychology and Theology*, 1983, 11(4), p. 340. English SWBS © 1982, C.W. Ellison & R.F. Paloutzian. Czech translated SWBS © 2015, R.F. Paloutzian. All rights reserved. Translation courtesy of Klara Malinakova and Peter Tavel. Not to be duplicated unless express written permission is granted by copyright holder

## Discussion

The aim of this study was to psychometrically evaluate the shortened version of the Spiritual Well-Being Scale in Czech adolescents. The first outputs of the internal consistency and factor analyses pointed to a problem with some of the items of the scale that created a separate factor. A closer inspection of this factor showed that it was formed by the three negatively formulated statements. Repeating the analyses after excluding those items was linked with a remarkable increase of the Cronbach’s alpha and Mean Inter-Item Correlation values and a reduction of the number of factors from original 3 to 2. Those correspond to the existing Religious and the Existential subscales of the SWBS.

Our results support the two-factorial model, consistent with the reports of the first study made by the authors of the scale (Ellison 1983) and other studies (Fernander et al. (2004); (Genia 2001). However, some other authors report in their studies three (Gow et al. 2011; Musa and Pevalin 2014) or more factors (Miller et al. 1998). The differences could be explained partly by the different cultural context regarding the expression of spirituality (Utsey et al. 2005). Moreover, factor analytic studies of the SWBS used various samples that differ in age, religiosity or education of respondents and also used different statistical techniques (Genia 2001; Musa and Pevalin 2012). Bufford et al. (1991) suggested that because of the ceiling effect the scores are negatively skewed, and so the assumptions of parametric correlational techniques are not met and factor analysis of the SWBS could lead to variable solutions.

We initially found a separate third factor consisting of the negatively worded items, which is consistent with the findings of other authors who studied negatively worded items. Several studies have shown that the factor analyses of scales containing both positive and negative items often reveal that an extra factor commonly emerges that is unique to the negatively worded items (DiStefano and Motl 2006). For example, Scott et al. (1998) describe in their study as a result of factor analysis 3 factors, one of which (Affiliation) consists of the positive items, while the two remaining (Alienation and Dissatisfaction with life) of the negative ones. Gow et al. (2011) also report three factors, the first one formed by 10 RWB items and 1 EWB item, while the second by the positive EWB items and a third one by the negative EWB items. Schmitt and Stults (1985) showed that even when only 10 % of respondents paid less attention to the wording, a separate factor loading on the negatively worded statements could be found. The study of Barnette (2000) reports that negatively worded items produce a lower Cronbach’s alpha. Including just a few negative items in an otherwise positively worded questionnaire seems to foster the tendency to misread the negative item because the person is being asked to shift a mental gear in processing the information (Roszkowski and Soven 2010). Taking into account that the SWBS used in our study was placed at the very end of the HBSC questionnaire, it might be possible that adolescents did not notice the reverse scoring of the items because of the tiredness and subsequent carelessness.

There is also another aspect that might have contributed to the confusion around the statement “I don’t get much personal strength and support from God” (item 5). As the focus group of older Czech adolescents, which was done in a consequent study, revealed, many of them chose the option “I totally disagree” as an expression of an overall disagreement with the question that speaks about the support from God they do not recognise. However, as a consequence of a negative formulation of the item their response was, after inversion, interpreted as a statement about maximal support. It may be that the negatively worded religious items lack the opportunity to express disagreement with the whole concept of the question that implicitly assumes the existence of God. Inasmuch as the majority of studies were performed on samples with distinctly higher rate of religious respondents, this study brings a new view on the usability of the SWBS in a secular environment.

### Strengths and Limitations

This study has several important strengths, the most important being the large and representative sample size of adolescents and the high response rate. It is also the first study that works with the shortened version of the SWBS in the Czech environment, a typical example of a secular society. On the other hand, a limitation of our study could be that due to incomplete data, which might have been caused by the length of the HBSC questionnaire battery, 13.7 % of questionnaires had to be excluded. Nevertheless, the proportion of religious/non-religious respondents corresponds to its prevalence in the population, so we might expect that our data are still representative. Another limitation might be an information bias, as our data were based on self-reports of adolescents, which can be inaccurate or influenced by social desirability. However, high levels of social desirability might be expected by religious respondents, who represent only 7.2 % of our sample. On the other hand, the wording of some items was not appropriate for non-believers, which might lead to information bias as well, e.g. “I don’t get much personal strength and support from God”. That item was finally the one with the highest shared loading and had to be excluded from the questionnaire.

### Implications

After removal of the negatively worded items, we found the 2-factor model of the shortened SWBS to be the most reliable. For adolescents in secular environment only this adjusted and shortened 7-item SWBS should be used. Generally, in this type of environment special attention should be paid to negative religious items where exclusion or some other way of dealing with the problem, e.g. including the option “does not apply to me”, should be considered. Further studies of religion and spirituality should consider that they are multidimensional constructs and that there might be an overlap with mental health (Koenig 2008). Using a scale that at the same time taps, e.g. mental or physical health should be avoided. Furthermore, it would be appropriate to compare samples of children with religious and secular attitudes.

## Conclusion

Our finding suggests that the adjusted shortened SWBS containing 7 items is suitable for measuring both religious and non-religious spirituality in a secularising environment. The adjusted, shortened SWBS (including 7 positively formulated items) might be a helpful instrument for future research.
